# Systematic analysis of concentrations of 52 elements in tumor and counterpart normal tissues of patients with non‐small cell lung cancer

**DOI:** 10.1002/cam4.2629

**Published:** 2019-10-23

**Authors:** Xin Cheng, Yong‐Chun Zhou, Bo Zhou, Yun‐Chao Huang, Gui‐Zhen Wang, Guang‐Biao Zhou

**Affiliations:** ^1^ State Key Laboratory of Molecular Oncology National Cancer Center/National Clinical Research Center for Cancer/Cancer Hospital Chinese Academy of Medical Sciences and Peking Union Medical College Beijing China; ^2^ State Key Laboratory of Membrane Biology Institute of Zoology Chinese Academy of Sciences and University of Chinese Academy of Sciences Beijing China; ^3^ Department of Thoracic Surgery The Third Affiliated Hospital of Kunming Medical University (Yunnan Tumor Hospital) Kunming China; ^4^ Beijing Institute of Genomics Chinese Academy of Sciences Beijing China; ^5^ Present address: Department of Cancer Biology Dana‐Farber Cancer Institute Boston MA 02215 USA

**Keywords:** concentrations, counterpart normal lung tissues, elements, lung cancer, tumor tissues

## Abstract

**Background:**

Many studies have documented the abnormal concentrations of major/trace elements in serum or malignant tissues of patients, but very few works systematically tested the concentrations of elements in tumor tissues in comparison with paired adjacent normal tissues from the same patients.

**Methods:**

Tumor and adjacent normal lung tissues were obtained from 93 patients with previously untreated NSCLC, and 43 patients whose tumor and paired normal lung tissues reached 200 mg or more were selected for measurement of the elements' concentrations using an inductively coupled plasma‐atomic emission spectrometer.

**Results:**

We found that the concentrations of the 52 elements varied from 0.4 ng/g tissue (Lu, Pd, and Tm) to 1 658 000 ng/g (Na), 1 951 000 ng/g (P), and 2 495 000 ng/g (K). Thirty eight of the 52 (73.1%) elements showed approximately equal concentrations in tumor and adjacent normal lung tissues of the patients. The concentrations of nine elements (K, P, Mg, Zn, Rb, Cu, Se, Cs, and Tl) in tumor samples were significantly higher than their paired normal lung tissues, and five elements (Na, Fe, Cr, Cd, and Ge) exhibited decreased concentrations in cancer samples compared to counterpart normal lung tissues. Low Fe in tumor samples was associated with smoking history, whereas low Cr was associated with histology (squamous cell carcinoma) of the patients.

**Conclusions:**

Our results demonstrate that measurement of elements’ concentrations in both cancer and paired normal tissues is important to get insights into the roles of these elements in carcinogenesis, and therapeutic approaches to normalize the elements are warranted to treat NSCLCs.

## Graphical abstract

By Systematic analysis of concentrations of 52 elements in tumor and counterpart normal tissues of patients with non‐small cell lung cancer, we showed that the concentrations of 14 elements (K, P, Mg, Zn, Rb, Cu, Se, Cs, Tl, Na, Fe, Cr, Cd, and Ge) were increased or decreased in lung tumor tissues compared to their counterpart normal lung tissues. These results indicate that normalization of these elements may have therapeutic potentials for NSCLCs.

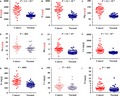

## 1 INTRODUCTION

Abnormalities in ion channels, the integral membrane proteins that allow the passive passage of certain ions into and out of the cell, have been linked to the development of cancers and immune evasion of cancer cells.^1^, ^2^ For example, alterations in Ca^2+^ channels such as the transient receptor potential (TRP) channels, the voltage‐gated Ca^2+^ channels (CACNAs) and Ryanodine Receptor (RYR) channels have been reported in some cancers^3^ including adenoid cystic carcinoma,^4^ Burkitt lymphoma,^5^ and lung cancer.^6^ Overexpression of *CACNA1E* is correlated with relapse in Wilms' tumors,^7^ while *CACNA2D1* plays a role in maintaining the properties of tumor‐initiating cells in hepatocellular carcinoma (HCC).^8^ Compared to normal cells, cancer cells usually have less negative membrane potential (*V*_m_) which is maintained mainly by K^+^ channels, and overexpression of K^+^ channels has been detected and linked to cancer hallmarks in several types of cancers.^1^ *KCNJ11* acts as an oncogene in HCC by forming a complex with lactate dehydrogenase A (LDHA).^9^ Abnormal Na^+^ and Cl^−^ channels also contribute to hallmarks of cancers.^1^ However, whether or not the alterations in ion channels result in abnormal ion levels in cancer cells compared to their adjacent normal counterparts remains to be investigated.

The roles of major/trace elements in cancers remain elusive. Serum levels of copper, an essential trace element that is necessary for the activity of a number of metalloenzymes,^10^ are elevated in Hodgkin's disease and correlate with the severity of the disease and response to therapies.^11^ People with higher serum copper, iron, or transferrin saturation concentrations have an increased risk of dying from cancer.^12^ However, another study showed that dietary copper as well as zinc intakes are associated with reduced risk of lung cancer.^13^ Serum selenium levels of cancer patients are significantly lower than that of healthy controls.^14^ Serum levels of Zn, Fe, and Mg were lower, and Cu, Mn, Ni, and Cr levels were higher in lung cancer patients than normal controls.^15^, ^16^ Malignant lung tissues from lung cancer patients had higher levels of Cu, Ca, Mg, and lower Zn than normal lung tissues from patients with nonmalignant diseases.^16^, ^17^, ^18^ However, very few studies systematically tested the concentrations of elements in tumor tissues in comparison with paired adjacent normal lung tissues from the same patients. Here, we aimed to clarify the element contents in cancers and their counterpart normal lung tissues in patients with non‐small cell lung cancer (NSCLC).

## 2 METHODS

### 2.1 Patient samples

The study was approved by the research ethics committees of our hospitals; all samples were collected with written informed consent from the patient's family, and were reviewed by two reference pathologists. Tumor and adjacent normal lung tissues (more than 5 cm away from tumors) were obtained from 93 patients with previously untreated NSCLC and were immediately frozen in liquid nitrogen after surgical resection. The tumor samples contained a tumor cellularity greater than 80% and the matched control samples had no tumor content. There were 43 patients whose tumor and paired normal lung tissues reached 200 mg or more (Table 1).

**Table 1:** The demographic characteristics of the 43 patients

Characteristics	Case, n (%)
Age, y	
≤59	26 (60.5)
60‐64	7 (16.3)
65‐69	5 (11.6)
≥70	5 (11.6)
Sex	
Female	13 (30.2)
Male	30 (69.8)
Smoking status	
Never	18 (41.9)
Former	2 (4.6)
Current	22 (51.2)
Unknown	1 (2.3)
Histology	
Adenocarcinoma	32 (74.4)
Squamous cell carcinoma	10 (23.3)
Not applicable	1 (2.3)
TNM stage	
I	18 (41.9)
II	5 (11.6)
III	10 (23.3)
IV	7 (16.3)
Not applicable	3 (6.9)

### 2.2 The elements

The 52 elements analyzed in this work were aluminum (Al), antimony (Sb), arsenic (As), barium (Ba), beryllium (Be), bismuth (Bi), cadmium (Cd), calcium (Ca), cerium (Ce), cesium (Cs), chromium (Cr), cobalt (Co), copper (Cu), dysprosium (Dy), erbium (Er), europium (Eu), gadolinium (Gd), germanium (Ge), holmium (Ho), iodine (I), iron (Fe), lanthanum (La), lead (Pb), lithium (Li), lutetium (Lu), manganese (Mn), magnesium (Mg), mercury (Hg), molybdenum (Mo), neodymium (Nd), nickel (Ni), palladium (Pd), phosphorus (P), platinum (Pt), potassium (K), praseodymium (Pr), rubidium (Rb), samarium (Sm), selenium (Se), silver (Ag), sodium (Na), strontium (Sr), terbium (Tb), thallium (Tl), thorium (Th), thulium (Tm), titanium (Ti), uranium (U), vanadium (V), ytterbium (Yb), yttrium (Y), and zinc (Zn).

### 2.3 Measurement of concentrations of the elements

The concentrations of the 52 elements were measured by Inductively Coupled Plasma‐Atomic Emission Spectrometer (ICP‐AES; Teledyne Leeman Labs) in 43/93 (46.2%) NSCLCs, whose tumor and adjacent normal lung tissues reached 200 mg or more. Equal amounts (200 mg) of tumor and paired normal lung tissues were ground in liquid nitrogen‐cooled mortar, the tissue powder was suspended in cold mixed acid (HNO_3_:HClO_4_, 1:1) overnight, and transferred to deionized water (5 mL). The concentrations of the elements were assessed by ICP‐AES using a multi‐element stock solution (Spex Instrumentation Industries). The certified reference material GBW08551 pork liver (from the National Institute of Standard Technology, Beijing, China) was used as the reference control sample.

### 2.4 Statistical analysis

All statistical analyses were conducted with GraphPad Prism 7 (GraphPad Software). The data are presented as the mean ± SD unless noted otherwise. Differences between data groups were evaluated for significance using a two‐sided Student's *t* test for paired samples. *P* values less than .05 indicate statistical significance.

## 3 RESULTS

### 3.1 The patients

Totally, 93 patients with NSCLC were involved in this study, including 43 patients whose isolated tumor and counterpart normal lung tissues were assessed for elements’ concentrations. The 43 patients were aged 55.4 years (range, 43.5‐79.6), 30 (69.8%) and 24 (55.8%) of them were male and smokers, respectively (Table 1).

### 3.2 The concentrations of the 52 elements

Using ICP‐AES analysis of paired samples of NSCLCs, we determined the concentrations of the elements in cancer and counterpart normal lung tissues of the patients (Table 2). We found that the concentrations of the 52 elements varied from 0.4 ng/g tissue (Lu, Pd, and Tm) to 1 658 000 ng/g (Na), 1 951 000 ng/g (P), and 2 495 000 ng/g (K). Thirty eight of the 52 (73.1%) elements showed approximately equal concentrations in tumor and adjacent normal lung tissues of the patients. These included Ag, Al, As, Ba, Be, Bi, Ca, Ce, Co, Dy, Er, Eu, Gd, Hg, Ho, I, La, Li, Lu, Mn, Mo, Nd, Ni, Pb, Pd, Pr, Pt, Sb, Sm, Sr, Tb, Th, Ti, Tm, U, V, Y, and Yb (Table 2). The concentrations of Ca in tumor and paired normal lung tissues were (142 900 ± 31 940) and (132 800 ± 28 780) ng/g (*P* = .82), respectively.

**Table 2:** Concentrations of the 52 elements in cancer and counterpart normal lung tissues of NSCLC patients

Element	Cancer tissue (ng/g)		Normal tissue (ng/g)		*P* values*
	Mean	SD	Mean	SD	
Ag	2.96	0.9591	13.51	7.409	.16
Al	62 480	15 590	69 920	10 110	.69
As	8.179	1.49	9.239	1.321	.6
Ba	184.5	40.77	245.1	31.31	.24
Be	1.826	0.4609	2.089	0.2814	.63
Bi	3.247	1.014	4.965	0.6991	.17
Ca	142 900	31 940	132 800	28 780	.82
Cd	236.4	29.95	437.4	62.13	.0046
Ce	149.9	42.99	153.7	21.03	.94
Co	33.07	8.719	40.56	4.544	.45
Cr	445	50.77	711.1	119	.04
Cs	28.37	2.521	21.98	1.615	.04
Cu	1518	82.29	1012	24.46	7.4 × 10^−8^
Dy	5.621	1.554	6.218	0.8172	.73
Er	3.112	0.8531	3.488	0.4466	.7
Eu	2.196	0.6693	2.275	0.3334	.92
Fe	131 400	19 980	223 600	17 910	.001
Gd	209.1	52.98	154	35.56	.39
Ge	19.66	2.76	31.86	2.548	.002
Hg	7.959	1.473	10.66	2.269	.32
Ho	1.069	0.2809	1.158	0.1513	.78
I	4251	2707	16190	12610	.36
K	2 495 000	97 700	1 598 000	32 610	2.3 × 10^−13^
La	82.82	24.08	82.26	11.42	.96
Li	36.47	6.974	37.93	4.666	.86
Lu	0.4221	0.1063	0.4177	0.05242	.97
Mg	123 400	4265	77 190	1570	2.6 × 10^−16^
Mn	249.2	24.19	247.7	23.72	.96
Mo	14.59	1.558	21.64	3.246	.054
Na	1 658 000	52 650	2 131 000	34 840	6.1 × 10^−11^
Nd	70.78	22.13	72.6	10.58	.94
Ni	1153	264.5	3339	2530	.4
P	1 951 000	74 710	1 169 000	32 160	3.6 × 10^−15^
Pb	49.87	7.731	139.6	60.09	.14
Pd	0.4488	0.1016	0.4795	0.0566	.79
Pr	18.31	5.432	18.63	2.662	.96
Pt	27.78	17.79	36.08	22.62	.77
Rb	6282	313.1	4121	164.6	3.0 × 10^−8^
Sb	20.56	11.17	17.85	3.602	.82
Se	286.5	23.13	182.5	13.21	.0002
Sm	10.69	3.429	10.65	1.595	.99
Sr	108.3	16.74	121.9	14.15	.54
Tb	1.154	0.3295	1.232	0.1658	.83
Th	10.47	2.692	11.77	1.555	.68
Ti	7850	2408	8373	1336	.85
Tl	5.707	0.8579	2.928	0.3256	.0033
Tm	0.39	0.1049	0.4253	0.05696	.77
U	3.177	0.8401	3.305	0.3875	.89
V	123.6	31.61	137.4	21.8	.72
Y	26.33	7.007	28.99	3.741	.74
Yb	3.59	0.6834	3.512	0.3987	.92
Zn	14450	588.4	12350	486.6	.01

* Tested using the two‐sided Student's *t* test for paired samples.

### 3.3 Elements exhibit higher concentrations in tumor samples

**Figure 1:**
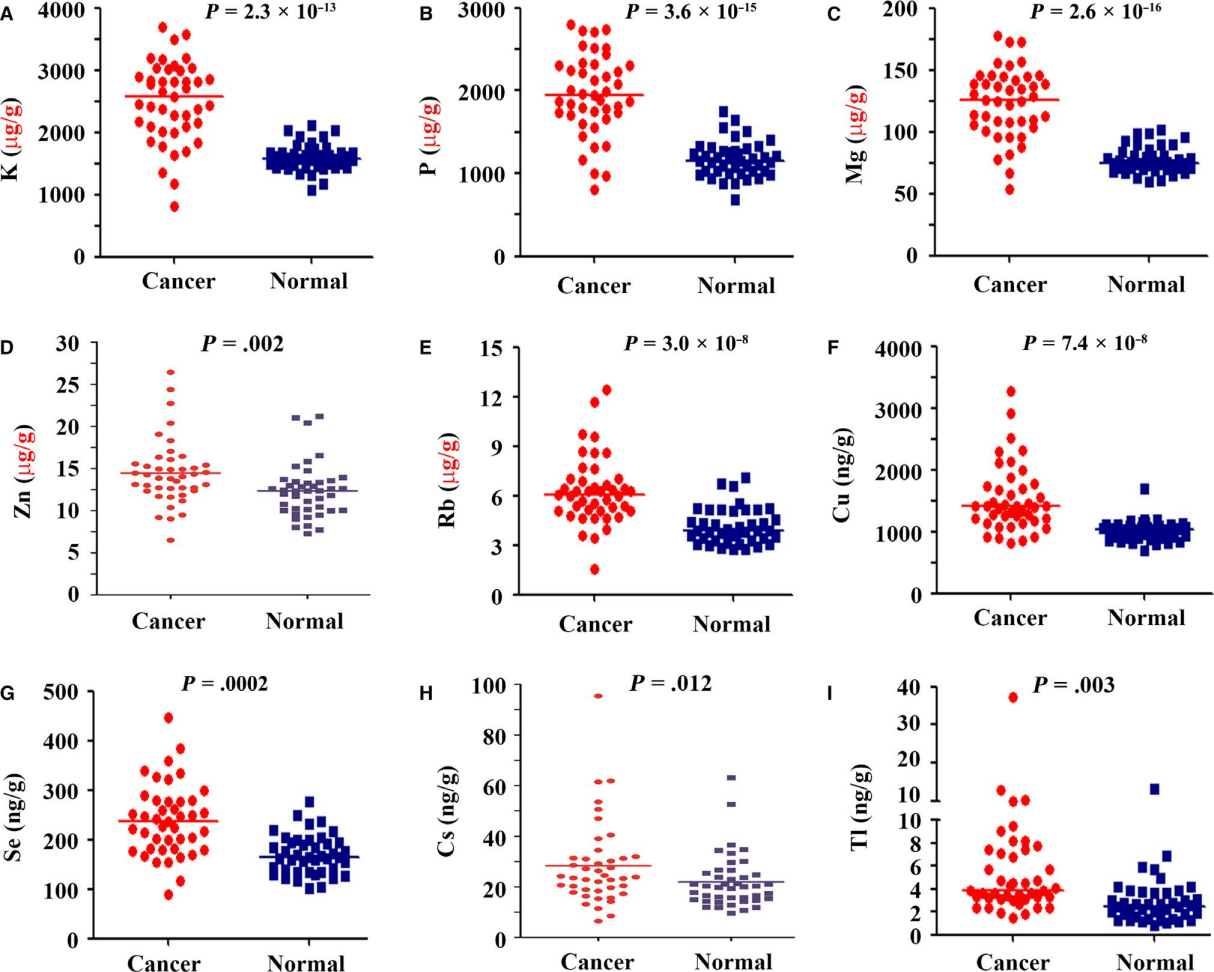
The nine elements that had higher concentrations in tumor tissues than in counterpart normal lung tissues. A‐I, The elements were K, P, Mg, Zn, Rb, Cu, Se, Cs, and Tl. P values were calculated using two‐sided Student's *t* test for paired samples

We showed that the concentrations of nine elements (K, P, Mg, Zn, Rb, Cu, Se, Cs, and Tl) in tumor samples were significantly higher than their paired normal lung tissues (Table 2; Figure 1). K was rich in lung tissues; the mean concentrations of K in tumor and normal lung tissues were 2 495 000 and 1 598 000 ng/g (*P* = 2.3 × 10^−13^), respectively. The contents of P, Mg, Zn, and Rb were also relatively high, and their concentrations in tumor samples were significantly higher than counterpart normal lung tissues (Figure 1). Cu, Se, Cs, and Tl showed higher concentrations in cancer tissues than paired normal lung samples (Figure 1). We analyzed the relationship between elevated elements and characteristics of the patients, and found that these elevations were not associated with any specific characteristics, including age, smoking history, TNM stages, and others.

### 3.4 Elements show higher concentrations in normal lung tissues

**Figure 2:**
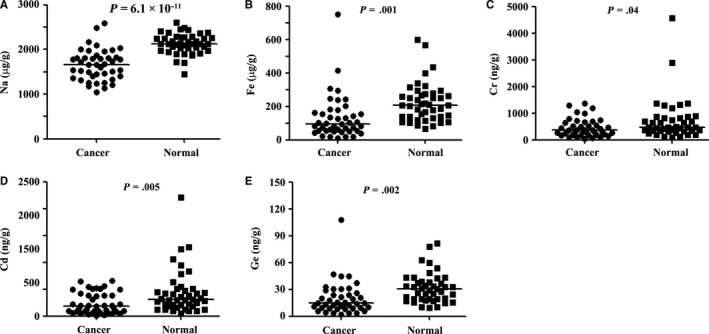
The five elements that had lower concentrations in tumor tissues than in counterpart normal lung tissues. A‐E, The elements were Na, Fe, Cr, Cd, and Ge. P values were calculated using the two‐sided Student's *t* test for paired samples

Five elements (Na, Fe, Cr, Cd, and Ge) exhibited decreased concentrations in cancer samples compared to counterpart normal lung tissues (Figure 2). Na was rich in normal lung tissues (2 131 000 ng/g tissue), and was decreased in counterpart cancer tissues (1 658 000 ng/g tissue; *P* = 6.1 × 10^−11^). Fe reached 223 600 ng/g in normal lung tissues, and was 131 400 ng/g in tumor samples (*P* = .001). Cr was (445 ± 50.77) and (711.1 ± 119) ng/g in cancer and paired normal lung tissues (*P* = .04), respectively. Cd and Ge also decreased in tumor samples as compared with counterpart normal lung tissues (Figure 2).

We analyzed the association between the concentration of the elements and characteristics of the patients, and found that low Fe was associated with smoking history (Table S1), in that low Fe was found in 23/25 (90%) smoker patients and in 11/17 (64.7%) nonsmoker patients (*P* = .03). Low Cr was found in 17/32 (53.1%) lung adenocarcinoma (LUADs) and in 10/10 (100%) lung squamous cell carcinoma (LUSCs) (*P* = .007; Table S2).

## 4 DISCUSSION

Concentrations of several trace and/or major elements in serum or tumor tissues of cancer patients have been tested in many works, but very few studies measured their concentrations in paired tumor and normal tissues. Observations in serum or tissues usually concluded that abnormal concentration of some elements (eg, elevated Fe and reduced Se) was associated with increased risk of cancer.^12^ However, by comparing with counterpart normal lung tissues, we showed that Fe concentration was decreased whereas K, Mg, P, Se, and Zn were increased in tumor tissues of NSCLCs (Table 2; Figures 1, 2). The causal correlationship between the element (Fe and Se, for example) content and cancer risk is complicated, and so far we did not know why concentrations of Fe and Se were perturbed in tumor samples. Further investigation should be done to determine whether abnormalities in these elements act as drivers in lung cancer initiation or only represent accompanying phenomena of NSCLC. Our results demonstrate that measurement of elements' concentrations in both cancer and paired normal tissues rather than only in serum or tumor tissues are important to get insights into the roles of these elements in carcinogenesis.

Some elements exert critical biological functions and are involved in cancer pathogenesis. For example, Fe is a vital micronutrient for human existence and plays a fundamental role in a wide range of cellular functions, including cellular proliferation, DNA synthesis, as well as DNA damage and repair, and has been strongly implicated in cancer development.^19^ The amount of iron absorbed by the intestine is tightly controlled to balance the daily losses. Cancer cells frequently have changes in the expression of iron regulatory proteins, eg, upregulation of transferrin (increasing uptake of iron) and downregulation of ferroportin (decreasing efflux of intracellular iron).^20^ We showed that Fe was decreased in tumor tissues compared to counterpart normal lung tissues (Table 2; Figure 1), confirming the observation that cancer cells exhibit an increased iron demand compared with normal, noncancer cells.^21^ Tobacco use is responsible for approximately 22% of all cancer deaths^22^ and more than 85% of lung cancer mortality worldwide.^23^ We showed that low Fe was seen in 23/25 (90%) smoker patients and in 11/17 (64.7%) nonsmoker patients (*P* = .03), suggesting that low Fe was associated with smoking history for unclear mechanisms.

Copper modulates oxidative phosphorylation and growth of tumors^24^ and is required for oncogenic BRAF signaling and tumorigenesis.^25^ We showed that Cu in tumor was significantly higher than in paired normal lung tissues [(1518 ± 82.29) vs (1012 ± 24.46) ng/g, *P* = 7.4 × 10^−8^], confirming that this element has a role in tumorigenesis. However, why copper was increased in tumor tissues remained unclear. Copper transporters CTR1/SLC31A1^26^ and CTR2/SLC31A2,^27^ and efflux transporters ATP7A^28^ and ATP7B,^29^ function to effect the uptake of dietary copper or the efflux of copper from cells respectively, and maintain cellular homeostasis of copper. The elevation of P in tumor tissues might reflect the active proliferation of cancer cells. The abnormalities in these genes should be unveiled to get insights into elevated copper in NSCLCs.

Se is a vital trace element involved in many biological processes that are mediated by selenoproteins.^30^ An inverse relation between Se exposure and cancer risk was suggested by early studies, but subsequent controlled trials reported that selenium supplementation does not reduce the risk of cancer and may even increase it for some cancer types.^31^ These observations may be explained by our findings that tumor tissues bear higher Se concentration than their paired normal lung tissues, and Se may have a role in promoting carcinogenesis. Zinc is an essential nutrient for human health, but its role in cancer prevention and treatment remains unclear.^32^ Magnesium (Mg^2+^) is also an essential ion to the human body.^33^ We showed that Zn, and Mg were significantly higher in tumor tissues than in their paired normal lung tissues, suggesting that these elements may be required by cancer cells and may play roles in promoting lung carcinogenesis. Cellular Mg^2+^ transporters SLC41A1 and CNNM4 function as Na^+^/Mg^2+^ exchangers, suggesting that Na may be reduced in tumor samples since Mg was elevated, and our results confirmed this possibility (Figure 2; Table 2).

## 5 LIMITATIONS

Our study has some limitations. First, the sample size is relatively small. Because the measurement requires a relatively large amount (200 mg) of both the cancer and normal lung tissues, only 43/93 (46.2%) met this requirement in this study. More paired samples should be detected in the future. Second, the mechanisms underlying the abnormalities of trace elements in cancers remain to be elucidated. Finally, the effects of normalization of the abnormal elements (ions) are not investigated in this study.

## 6 CONCLUSIONS

Targeting abnormal trace elements has emerged as a novel therapeutic strategy for cancers. For example, copper depletion exhibits promising efficacy in pre‐clinical animal models and in a phase I clinical trial,^34^ and ferumoxytol displays anti‐leukemia efficacy against cells with low ferroportin levels.^35^ Copper chelating agents and ferumoxytol also show anti‐lung cancer activity in preclinical studies.^36^, ^37^ We showed that the concentrations of 14 elements were increased or decreased in lung tumor tissues compared to their counterpart normal lung tissues, suggesting that normalization of these elements may have therapeutic potentials for NSCLCs. Therefore, further studies are needed to develop therapeutic approaches to normalize the elements to treat NSCLCs.

## CONFLICT OF INTEREST

No potential conflicts of interest were disclosed.
