# Supplementary material for: Systematic analysis of concentrations of 52 elements in tumor and counterpart normal tissues of patients with non‐small cell lung cancer
Source: Cancer Med. 2019 Oct 23;8(18):7720–7. doi: 10.1002/cam4.2629 (PMC6912044; doi:10.1002/cam4.2629)
Supplement: Supplementary file 1 [file CAM4-8-7720-s001.docx]

**Supplementary information**

**Zhao et al.**

**Systematic analysis of concentrations of 52 elements in tumor and counterpart normal tissues of patients with non-small cell lung cancer**

Table S1. Low Fe concentration and patient characteristics.

| **Characteristics** | **Total cases (n=43)** | **Fe-low, n (%)** | **P values*** |
| --- | --- | --- | --- |
| Gender |  |  |  |
| Male | 29 | 25 (86.2) | 0.2 |
| Female | 13 | 9 (69.2) |  |
| Unknown | 1 | 1 |  |
| Age |  |  |  |
| <65 | 34 | 29 (85.3) | 0.14 |
| ≥65 | 8 | 5 (62.5) |  |
| Unknown | 1 | 1 |  |
| Smoking history |  |  |  |
| Smoker | 25 | 23 (92) | 0.03 |
| Non-smoker | 17 | 11 (64.7) |  |
| Unknown | 1 | 1 |  |
| Histology |  |  |  |
| Adenocarcinoma | 32 | 25 (78.1) | 0.4 |
| Squamous cell carcinoma | 10 | 9 (90) |  |
| Unknown | 1 | 1 |  |
| TNM stage |  |  |  |
| I | 19 | 16 (84.2) | 0.31 |
| II | 5 | 5 (100) |  |
| III | 9 | 6 (66.7) |  |
| IV | 7 | 6 (85.7) |  |
| Unknown | 3 | 2 |  |

* tested by the Fisher exact test.

Table S2. Low Cr concentration and patient characteristics.

| **Characteristics** | **Total (n=43)** | **Cr-low, n (%)** | **P values** |
| --- | --- | --- | --- |
| Gender |  |  |  |
| Male | 29 | 20 (69) | 0.34 |
| Female | 13 | 7 (53.8) |  |
| unknown | 1 | 1 |  |
| Age |  |  |  |
| <65 | 34 | 23 (67.6) | 0.35 |
| ≥65 | 8 | 4 (50) |  |
| unknown | 1 | 1 |  |
| Smoking |  |  |  |
| Smoker | 25 | 18 (72) | 0.21 |
| Non-smoker | 17 | 9 (52.9) |  |
| unknown | 1 | 1 |  |
| Histology |  |  |  |
| Adenocarcinoma | 32 | 17 (53.1) | 0.007 |
| Squamous cell carcinoma | 10 | 10 (100) |  |
| unknown | 1 | 1 |  |
| TNM stage |  |  |  |
| I | 19 | 12 (63.2) | 0.34 |
| II | 5 | 5 (100) |  |
| III | 9 | 6 (66.7) |  |
| IV | 7 | 3 (42.9) |  |
| unknown | 3 | 2 |  |

* tested by the Fisher exact test.
